# PD-L1 Expression and Immune Microenvironment Remodeling During Laryngeal Carcinogenesis: Implications for Malignant Progression of Laryngeal Dysplasia

**DOI:** 10.3390/ijms27167195

**Published:** 2026-08-12

**Authors:** Filip Tudor, Blažen Marijić, Emina Babarović, Ita Hadžisejdić

**Affiliations:** 1 Department of Otorhinolaryngology, Head and Neck Surgery, Clinical Hospital Center Rijeka, 51000 Rijeka, Croatia; filip.tudor@uniri.hr (F.T.); blazen.marijic@uniri.hr (B.M.); 2 Faculty of Medicine, University of Rijeka, 51000 Rijeka, Croatia; emina.babarovic@medri.uniri.hr; 3 Clinical Department of Pathology and Cytology, Clinical Hospital Center Rijeka, 51000 Rijeka, Croatia

**Keywords:** laryngeal dysplasia, malignant transformation, PD-L1, tumor-associated macrophages, tumor microenvironment

## Abstract

Laryngeal dysplasia (LD) is the principal precursor of laryngeal squamous cell carcinoma (LSCC), but reliable biomarkers predicting malignant transformation remain unavailable. This study investigated programmed death-ligand 1 (PD-L1) expression and immune microenvironment remodeling during laryngeal carcinogenesis. Immunohistochemical analysis of PD-L1, CD4, CD8, CD68, and CD163 was performed in 36 benign lesions, 41 LDs, and 102 LSCCs. PD-L1 expression was assessed using the combined positive score (CPS) and tumor proportion score (TPS), while immune cell infiltration was quantified in the intraepithelial and stromal compartments and across the entire tissue specimen. PD-L1 expression and infiltration of all investigated immune cell populations increased progressively from benign lesions through dysplasia to invasive carcinoma. High-grade dysplasia exhibited significantly greater CD4 infiltration in all compartments and increased stromal CD8 and CD163 infiltration than low-grade dysplasia. PD-L1 expression positively correlated with intraepithelial CD163 and stromal CD8 infiltration. Although no biomarker independently distinguished progressive from non-progressive dysplasia, overall CD4, intraepithelial and overall CD68, and stromal CD163 were associated with progression risk. Immune microenvironment remodeling begins during the dysplastic stage of laryngeal carcinogenesis. These findings may improve risk stratification of laryngeal dysplasia and support the development of immunopreventive and immune-targeted therapeutic strategies.

## 1. Introduction

For laryngeal dysplasia (LD), several noteworthy attempts have been made over the last sixty years to establish a generally accepted terminology and histological grading system [1]. Relatively recent World Health Organization (WHO) classification (2017) suggests a two-tier approach with rather strict histological criteria, dividing dysplasia into low- and high-grade categories [1,2]. According to published data, malignant transformation occurs in approximately 5–10% of low-grade lesions and 15–30% of high-grade lesions, with increasing risk of transformation depending on severity of dysplasia [2,3,4]. There are no clear morphological indicators as to whether any of the precancerous lesions will progress to laryngeal squamous cell cancer (LSCC) and, if so, during what period. The therapy and follow-up of LD is challenging because each new biopsy results in tissue scarring, which subsequently causes voice abnormalities. Furthermore, a more severe therapeutic strategy may be necessary depending on the extent of the dysplasia, which could result in permanent hoarseness. On the other hand, histological diagnosis is significantly influenced by subjectivity, uncertainty and interobserver variability. Moreover, the existence of multiple classification systems that were employed concurrently in various institutions impeded data comparison and delayed the development of clear guidelines and consensus. Based on the previously mentioned data, it is evident that novel markers are required to predict the progression of LD into LSCC. Therefore, it is of utmost significance to identify supplementary morphological, immunohistochemical or molecular indicators that would facilitate a precise evaluation of the precancerous lesions that are likely to progress into cancer.

Increasing evidence indicates that carcinogenesis is influenced not only by genetic alterations within epithelial cells but also by dynamic interactions between transformed cells and the surrounding immune microenvironment. During malignant progression, dysplastic cells gradually acquire mechanisms that enable them to evade immune surveillance, thereby promoting their survival and progression toward invasive carcinoma. Understanding these early immune alterations is therefore of particular interest, as they may improve risk stratification of premalignant lesions and identify potential targets for preventive therapeutic strategies.

One of the principal mechanisms of tumor immune escape is activation of the programmed death-1/programmed death-ligand 1 (PD-1/PD-L1) immune checkpoint pathway. PD-L1 is a type I transmembrane glycoprotein expressed by epithelial cells, immune cells, and malignant cells, where its interaction with PD-1 suppresses T-cell activation and antitumor immune responses [5]. In addition to immune checkpoint signaling, the tumor microenvironment, comprising immune and stromal cells, plays a central role in regulating tumor development, immune surveillance, and immune evasion.

Among immune cell populations, CD8+ cytotoxic T lymphocytes represent the major effector cells responsible for direct antitumor immunity, whereas CD4+ T lymphocytes regulate adaptive immune responses through both helper and regulatory subsets. Macrophages constitute another essential component of the tumor microenvironment. CD68 is widely used as a marker of the overall macrophage population, whereas CD163 identifies alternatively activated (M2-like) tumor-associated macrophages that promote immunosuppression, tissue remodeling, angiogenesis, and tumor progression. Simultaneous assessment of these immune cell populations together with PD-L1 expression therefore provides a comprehensive overview of immune microenvironment remodeling during laryngeal carcinogenesis.

In head and neck squamous cell carcinoma (HNSCC), including laryngeal squamous cell carcinoma, advances in immuno-oncology have established the PD-1/PD-L1 pathway as an important therapeutic target. Immune checkpoint inhibitors have expanded treatment options for patients with recurrent, persistent, or metastatic disease [6], highlighting the critical role of tumor–immune system interactions. It is considerably less known about activation of this pathway during the premalignant stages of laryngeal carcinogenesis.

Although the PD-1/PD-L1 pathway has been extensively investigated in established HNSCC, its role during the early stages of laryngeal carcinogenesis remains poorly understood [7,8,9]. Since only a subset of laryngeal dysplasias progress to invasive squamous cell carcinoma, identifying early immunological alterations associated with malignant transformation is of considerable clinical importance [10]. Evaluation of PD-L1 expression together with immune cell infiltration may therefore provide valuable insight into immune escape mechanisms before invasion and may facilitate identification of biomarkers associated with progression risk.

There is a lack of studies comparing expression of PD-L1 and tumor microenvironment in precancerous lesions that progressed into cancer with those that do not. Previous studies investigating precancerous lesions of the oral cavity have demonstrated that the expression of PD-L1 was found to be significantly elevated in precancerous lesions that exhibited progression, as compared to those that did not progress [11].

Thus, the objectives of this research were as follows: (i) to evaluate the expression level of PD-L1 and infiltration by CD4, CD8, CD68 and CD163 cells in benign laryngeal lesions, low and high-grade LD and LSCC; (ii) to assess a correlation between PD-L1 expression and tumor microenvironment cells (CD4, CD8, CD68, CD163) in LD; (iii) to compare the expression level of PD-L1 and tumor microenvironment cells (CD4, CD8, CD68, CD163) in LD that progressed to LSCC as opposed to those that did not.

To our knowledge, this is one of the first studies to comprehensively evaluate PD-L1 expression together with CD4+, CD8+, CD68+, and CD163+ immune cell infiltration across the entire spectrum of laryngeal carcinogenesis, while also comparing progressive and non-progressive laryngeal dysplasia. This approach provides novel insight into immune microenvironment remodeling during early laryngeal carcinogenesis and may contribute to the identification of biomarkers associated with malignant transformation.

## 2. Results

### 2.1. PD-L1 Expression and CD4, CD8, CD68 and CD163 Infiltrate in Benign Lesions, LD and LSCC

The greatest differences in investigated markers between LD and LSCC were observed for stromal and overall CD4 as well as stromal and overall CD163 infiltration. Similarly, the largest differences between the LD and control groups were found for stromal and overall CD163 and overall CD8 infiltration. Post hoc analysis was performed to identify pairwise differences between the groups in which statistically significant differences were detected. For most analyzed parameters, significant differences were found among the control, LD and LSCC groups. However, post hoc analysis revealed that intraepithelial CD4 and CD8 expression increased already at the stage of dysplasia, whereas stromal CD68, overall CD68, and overall CD163 expression increased only at the stage of invasive squamous cell carcinoma. Data are shown in Table 1.

### 2.2. A Comparison of PD-L1 Expression and CD4, CD8, CD68 and CD163 Infiltration in Low-Grade Versus High-Grade LD

**Figure 1:**
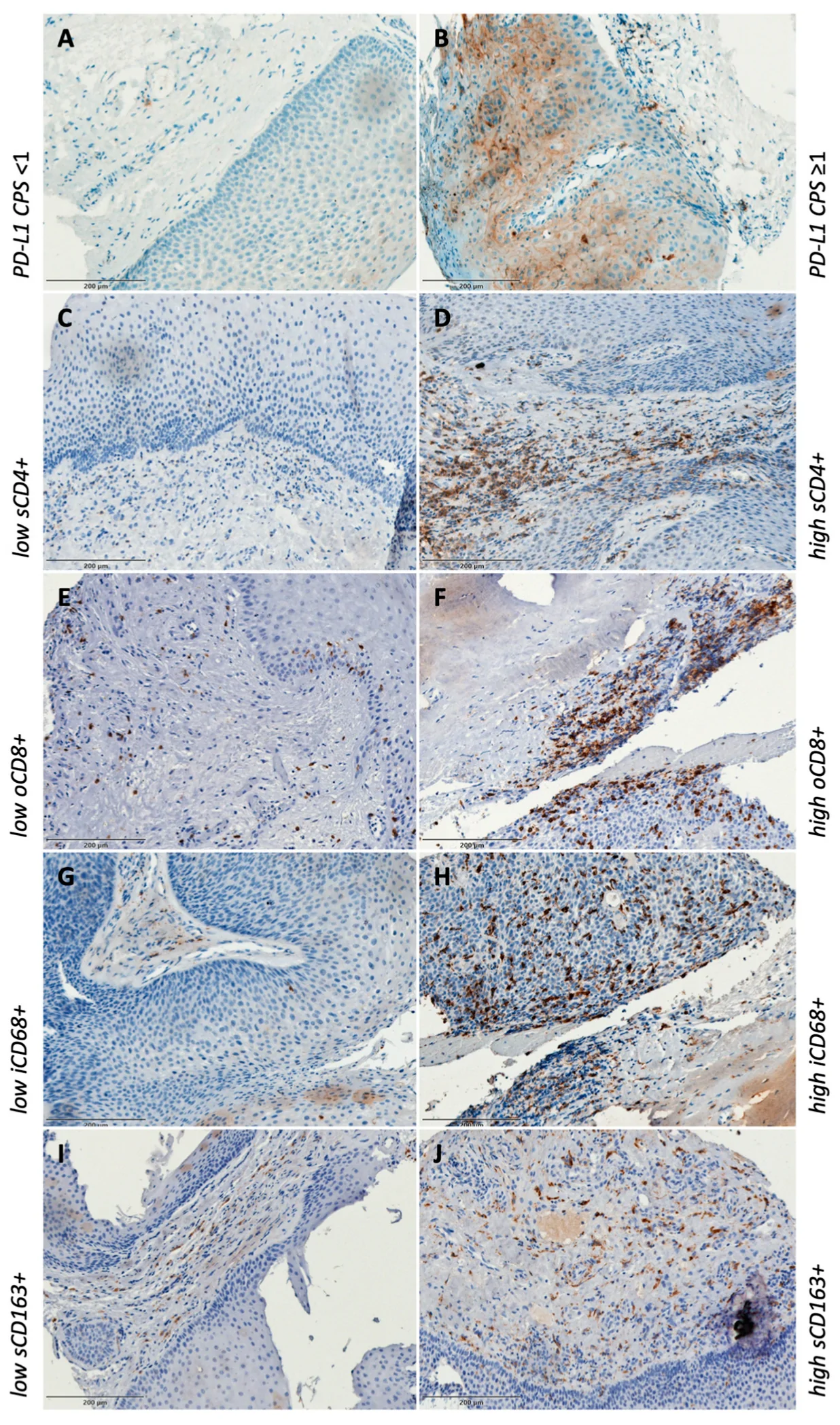
Immunohistochemical staining of PD-L1-negative and -positive CPS (**A**,**B**) and low and high infiltration for CD4+ (**C**,**D**), CD8+ (**E**,**F**), CD68+ (**G**,**H**) and CD163+ (**I**,**J**). s—stromal; o—overall; and i—intratumoral. Abbreviations: CPS—combined positive score.

Compared with low-grade dysplasia, high-grade dysplasia was associated with significantly increased infiltration of CD4-positive cells in all compartments analyzed (intraepithelial, stromal, and overall; *p* = 0.025, *p* = 0.002, and *p* = 0.003, respectively). In addition, stromal infiltration of CD8-positive and CD163-positive cells was significantly higher in high-grade lesions (*p* = 0.019 and *p* = 0.015, respectively). Although not reaching statistical significance, high-grade dysplasia also showed trends toward increased overall CD8 infiltration, stromal and overall CD68 infiltration, and CPS median expression (*p* = 0.066, *p* = 0.079, *p* = 0.078, and *p* = 0.061, respectively) (Table 2). Figure 1 shows examples of immunohistochemical staining of the investigated markers in laryngeal dysplasia.

### 2.3. Correlation of PD-L1 Expression with CD4, CD8, CD68 and CD163 Infiltration in LD

Both CPS and TPS PD-L1 expression scores showed a statistically significant positive correlation with intraepithelial CD163 infiltration (r_s_ = 0.356, *p* = 0.049 and r_s_ = 0.439, *p* = 0.013, respectively). Furthermore, there was statistically significant positive correlation between CPS and CD8 stromal compartment (r_s_ = 0.402, *p* = 0.02), while TPS and CD8 stromal compartment showed results at the level of statistical trend (r_s_ = 0.323, *p* = 0.066) (Table 3).

### 2.4. Expression of PD-L1 and CD4, CD8, CD68 and CD163 Infiltration in LD That Progressed to Carcinoma in Comparison to LD That Did Not Progress

When comparing precancerous lesions that progressed to carcinoma with those that did not, there was no statistically significant difference in any of the investigated markers. However, higher infiltration of CD163 cells in the stromal compartment was found in LD that progressed to LSCC (*p* = 0.07) but the result was at the level of statistical trend. On the other hand, higher infiltration of CD68 cells in the intraepithelial compartment was found in LD that did not progress to LSCC (*p* = 0.078), also at the level of statistical trend. Data are shown in Table 4.

### 2.5. Factors Associated with LD Progression

Statistically significant factors of LD progression were overall CD4 infiltration (cut-off > 6, *p* = 0.034, AUC = 0.694), intratumoral CD68 infiltration (cut-off ≤ 0.5, *p* = 0.043, AUC = 0.709), overall CD68 infiltration (cut-off ≤ 3, *p* = 0.043, AUC = 0.709), and stromal CD163 infiltration (cut-off > 7.5, *p* = 0.020, AUC = 0.729) (Table 5). Conversely, low CD68 expression (intratumoral ≤ 0.5 and overall ≤ 3) was associated with significantly reduced odds of progression (OR = 0.161, 95% CI: 0.03–0.95, *p* = 0.043 for both), suggesting a potential protective effect.

## 3. Discussion

Histological grading of laryngeal dysplasia has remained the most significant prognostic factor for predicting the biological behavior of disease and for directing clinicians in choosing the most effective approach to treatment [12]. However, the question arises if this is enough in the age of precision medicine?

Depending on the severity, dysplasia progresses to LSCC in 0 to 48% of cases. But since we are unable to distinguish between potentially progressive lesions and those that are not, the treatment is the same and frequently has unwanted consequences on laryngeal functions because of a more aggressive approach. Regretfully, up till now, no precise morphological criteria or biomarker has been developed to differentiate potentially progressive lesions from non-progressive ones. Therefore, it is expected that research efforts in science are concentrated on identifying possible indicators of progression. In the past few decades, a great deal of interest has been given to the immune cells that make up the microenvironment and their relationship to the PD-L1 molecule.

PD-L1 is a functionally described ligand for co-inhibitory PD-1, sometimes referred to as B7-H1 or CD274. In the last few years, the PD-1/PD-L1 co-inhibitory pathway has shown potential as a target for cancer therapy. Numerous studies have demonstrated usage of anti-PD-L1 antibody medications in clinical settings and that PD-L1 expression levels are adverse prognostic markers with unfavorable clinical outcomes, including reduced overall survival and increased disease progression, in various malignancies [13,14]. While the PD1/PD-L1 pathway has been used in the field of oncology, our understanding of its involvement in preinvasive lesions in general, particularly in laryngeal preinvasive lesions, remains limited.

The primary objectives of our study were to assess the levels of PD-L1 expression across different stages of carcinogenesis, ranging from benign lesions to low and high-grade LD, and ultimately to LSCC. We identified a significant difference between these groups (*p* < 0.0001). The findings of a recent meta-analysis conducted by Girolami et al. suggest that PD-L1 expression is more common in precancerous lesions compared to normal mucosa (risk ratio [RR] 1.65, CI 0.65–4.03, I^2^ 91%, tau^2^ 0.82), and less common than in invasive squamous cell carcinoma (RR 0.68, CI 0.43–1.08, I^2^ 91%, tau^2^ 0.22) [15]. These results line up with our findings, but it is worth noting that most of the studies included in this analysis relate to oral precancerous lesions. Based on the same study, the prevalence of PD-L1 in dysplasia was 45.04% (CI 7.39–86.30, I^2^ 98%, tau^2^ 0.2936), which is consistent with our data that indicate a CPS > 1 in 34.3% (*n* = 12/35) of dysplasia cases. It is worth mentioning that PD-L1 expression is less prominent in healthy laryngeal mucosa compared to precancerous lesions, also supported by other research data [16,17,18]. Our findings further support this, as only 3.6% of patients in the control group (*n* = 1/28) had CPS > 1. In addition, the study revealed a slightly greater frequency of CPS expression in high-grade dysplasia compared to low-grade dysplasia (36.4% vs. 21.1%, respectively). However, we were unable to establish statistical significance when comparing these lesions, except when we compared median CPS PD-L1 expressions, which showed a statistical trend (*p* = 0.061). It is possible if a greater number of cases were analyzed, significant differences would be observed. Regrettably, we were unable to identify differences in CPSs and TPSs between the lesions that progressed into SCC and those that did not. Nevertheless, the progressive increase in PD-L1 expression observed during laryngeal carcinogenesis suggests that activation of the PD-1/PD-L1 axis may occur before invasive carcinoma develops. Although immune checkpoint inhibitors are currently reserved for advanced HNSCC, these findings raise the possibility that PD-L1 expression could contribute to future risk stratification strategies and support investigation of immunomodulatory approaches in selected high-risk premalignant lesions. If validated in larger prospective studies, such approaches could significantly alter the management of precancerous laryngeal lesions, particularly in patients with extensive disease, multifocal lesions, or dysplasia at surgical margins, while potentially reducing progression to invasive squamous cell carcinoma. However, these potential clinical applications require validation in larger prospective studies.

It is evident that dysplastic cells use several tactics in their progression to cancer, such as escaping immune-mediated elimination. For this reason, our study also included immune microenvironment cells and analyzed the presence and density of CD4+, CD8+, CD68+, and CD163+ cells overall, as well as separately within the stromal and intraepithelial compartments.

The results demonstrated a statistically significant increase in the expression of all investigated cell types from the control group, through LD to LSCC. In other words, the lowest expression levels were observed in benign lesions, whereas the highest expression was found in squamous cell carcinoma. This pattern was observed in the overall analysis as well as when the epithelial and stromal compartments were evaluated separately (Table 1), which is consistent with previous studies investigating oral mucosal lesions [16,19]. Moreover, the expression of the investigated immune cell populations was significantly higher in high-grade dysplasia compared with low-grade dysplasia, particularly for CD4+ cells, both in the overall analysis and within the intraepithelial and stromal compartments. In contrast, statistically significant differences for CD8+ and CD163+ cells were observed only within the stromal compartment.

Correlation analysis between PD-L1 expression and immune microenvironment components revealed a positive and statistically significant association between PD-L1 expression (assessed using CPS and TPS) and CD163+ cells in the intraepithelial compartment, as well as between PD-L1 expression (assessed using CPS) and CD8+ cells in the stromal compartment. Due to the limited number of studies investigating these markers in laryngeal dysplasia, our findings were compared with previously reported data from oral premalignant lesions. In the study by Stasikowska-Kanicka et al., a statistically significant negative correlation between PD-L1-positive cells and CD8-positive cells was observed in oral carcinoma, while a positive correlation between PD-L1-positive cells and CD163-positive cells was reported in both dysplasia and carcinoma groups, findings that are partly consistent with our results [19].

One of the most important findings of the present study was the positive correlation between PD-L1 expression and CD163+ macrophage infiltration. CD163 is widely recognized as a marker of alternatively activated (M2-like) macrophages, which contribute to an immunosuppressive microenvironment through secretion of IL-10, TGF-β, and other immunomodulatory mediators [20]. Conversely, inflammatory cytokines, particularly interferon-γ (IFN-γ), can induce PD-L1 expression on both tumor cells and macrophages. These observations suggest a bidirectional interaction in which M2-like macrophages and PD-L1 signaling reinforce each other, promoting immune escape during laryngeal carcinogenesis [21]. Although the cross-sectional design of the present study precludes conclusions regarding causality, our findings support the concept that macrophage polarization and immune checkpoint activation are closely linked during disease progression.

Similarly, Yagyu et al. demonstrated that increased expression of subepithelial CD163-positive cells, together with increased epithelial and subepithelial PD-L1 expression, was significantly associated with malignant transformation of oral epithelial dysplasia [22]. Using multivariate logistic regression analysis, the authors further showed that an increased number of subepithelial CD163-positive cells was significantly associated with the presence of high-grade dysplasia, independently of the number of subepithelial CD8-positive cells, PD-L1 expression in epithelial and subepithelial compartments, and lesion location. These findings support our observation of a significantly higher expression of CD163-positive cells in high-grade dysplasia. This phenomenon suggests that increasing severity of epithelial dysplasia is accompanied by enhanced recruitment of tumor-associated macrophages (TAMs) to the superficial lamina propria [23]. Such recruitment may be driven by dysplastic epithelial cells through chemokine signaling, including colony-stimulating factor-1 (CSF-1) [23,24]. Tumor-associated macrophages are widely recognized as key mediators of an immunosuppressive tumor microenvironment and may therefore contribute to the progression of epithelial dysplasia and early carcinogenesis [25,26].

CD4+ tumor-infiltrating lymphocytes are generally considered to play a protective role in antitumor immunity, and increased CD4+ infiltration has often been associated with improved clinical outcomes and better survival [27]. Our finding that overall CD4+ cell density > 6 was associated with an increased risk of progression appears to contrast with this traditional view. However, CD4+ T-cell populations are functionally heterogeneous and include not only effector helper T-cells but also immunosuppressive regulatory T-cells (Tregs), which suppress cytotoxic CD8+ T-cell activity and promote immune tolerance within the tumor microenvironment. An increased proportion of Tregs has been associated with immune escape and disease progression in HNSCC, suggesting that the prognostic impact of CD4+ infiltration may depend more on the balance of CD4+ subpopulations than on the total number of CD4+ cells [28,29]. Because our study evaluated total CD4+ cells without distinguishing functional subsets, further studies incorporating markers such as FOXP3 are needed to clarify which CD4+ populations drive malignant progression.

In the present study, no statistically significant differences in the expression of the investigated markers were observed between progressive and non-progressive dysplasias. Although stromal expression of CD163-positive cells was higher in dysplasias that progressed to carcinoma, this finding reached only the level of a statistical trend (*p* = 0.07).

The present study expands the current understanding of immune microenvironment changes during laryngeal carcinogenesis by simultaneously evaluating PD-L1 expression and multiple immune cell populations (CD4+, CD8+, CD68+, and CD163+) across benign lesions, low-grade dysplasia, high-grade dysplasia, and invasive carcinoma. In addition, unlike most previous studies, we specifically compared progressive and non-progressive laryngeal dysplasia, addressing an important gap in the current literature. Although the limited number of progression events precluded definitive conclusions regarding predictive biomarkers, our findings provide a basis for future prospective studies investigating immune-based risk stratification of laryngeal dysplasia.

The main limitation of the present study is the relatively small number of patients with progressive laryngeal dysplasia. Consequently, the statistical power to detect differences between progressive and non-progressive lesions was limited, increasing the possibility of Type II error. Therefore, the absence of statistically significant associations for several investigated biomarkers should be interpreted with caution and should not be considered evidence of a lack of biological relevance. Future multicenter studies including larger cohorts of progressive dysplasia are warranted to validate these findings.

## 4. Materials and Methods

### 4.1. Study Cohort

This retrospective study was conducted using samples obtained from the archives of the Clinical Department of Pathology and Cytology, Clinical Hospital Center Rijeka. The biopsy samples included in this study were obtained from the patients who were treated for laryngeal lesions at the Clinic for Otorhinolaryngology and Head and Neck Surgery, Clinical Hospital Center Rijeka between 2010 and 2019. All the patients were primarily surgically treated. The cohort included 102 patients diagnosed with LSCC, 41 patients with LD and 36 patients with benign laryngeal lesions (vocal fold polyps and nodules), who served as the control group. Histopathological examination confirmed the absence of epithelial dysplasia in all control specimens. These lesions were selected as controls because they are benign, non-neoplastic conditions without malignant potential, thereby providing an appropriate reference for comparison with dysplastic and malignant lesions. As normal laryngeal mucosa from healthy individuals is rarely available for ethical and practical reasons, benign surgical specimens are commonly used as reference tissue in studies investigating laryngeal carcinogenesis. Information on patient’s age at the time of the first diagnosis, the grade of LD, and the time to progression were obtained from the patient’s medical records. The latest (WHO 2017) grading system was utilized for LD classification [1,12,30].

### 4.2. Immunohistochemistry

Immunohistochemical staining was performed on 3–4 μm sections cut from tissue microarray (TMA) paraffin blocks and mounted on positively charged glass slides. PD-L1 expression was assessed using the rabbit monoclonal antibody SP263 (Ventana Medical Systems, Tucson, AZ, USA; ready-to-use). CD4, CD8, CD68, and CD163 were detected using rabbit monoclonal anti-CD4 (clone SP35, Cell Marque, Rocklin, CA, USA; dilution 1:75), mouse monoclonal anti-CD8 (clone C8/144B; ready-to-use), mouse monoclonal anti-CD68 (clone PG-M1, Dako Agilent, Santa Clara, CA, USA; dilution 1:100), and mouse monoclonal anti-CD163 (clone MRQ-26, Cell Marque, Rocklin, CA, USA; dilution 1:25). For PD-L1, heat-induced epitope retrieval was performed in Cell Conditioning 1 (CC1; Tris-based buffer, pH 8.0–8.5) for 64 min using the BenchMark Ultra automated staining platform (Ventana Medical Systems, Tucson, AZ, USA). For CD4, CD8, CD68, and CD163, antigen retrieval was performed using Target Retrieval Solution, high pH (Tris/EDTA, pH 9; Dako Agilent, Glostrup, Denmark) in the PT Link system (Agilent Technologies, Santa Clara, CA, USA) for 20 min at 97 °C. PD-L1 immunostaining was performed on the BenchMark Ultra platform according to the manufacturer’s instructions, whereas CD4, CD8, CD68, and CD163 staining was carried out using the Dako Autostainer Plus (DakoCytomation, Fort Collins, CO, USA). Immunoreactivity was visualized using the OptiView DAB IHC DetDako Agilent, Santa Clara, CA, USAection Kit (Ventana Medical Systems, Tucson, AZ, USA) for PD-L1 and the EnVision™ Detection System, Rabbit/Mouse (Dako REAL™, Glostrup, Denmark) for CD4, CD8, CD68, and CD163, with 3,3′-diaminobenzidine (DAB) as the chromogen. Slides were counterstained with hematoxylin. Negative controls were prepared by replacing the primary antibody with buffer solution.

### 4.3. Evaluation of Immunoreactivity

The independent evaluation of the expression of investigated biomarkers was conducted by two pathologists who were blinded for the patients’ follow-up data. Combined positive score (CPS) and tumor proportion score (TPS) were used to assess the level of PD-L1 expression, where CPS < 1 and TPS < 1 indicated negative expression and CPS ≥ 1 and TPS ≥ 1 indicated positive expression. The CPS was calculated as follows: the number of PD-L1-positive cells including tumor cells, macrophages and lymphocytes was divided by the total number of viable tumor cells and then multiplied by 100 [31]. TPS was calculated by the number of PD-L1-positive tumor cells divided by the total number of all viable tumor cells and then multiplied by 100 [32]. TMA cores that contained fewer than 100 viable tumor cells were excluded.

The assessment methodology used for CD4, CD8, CD68 and CD163 was derived from the Guidelines for the Assessment of Tumor-Infiltrating Lymphocytes (TILs) in Solid Tumors: Recommendations by an International Immuno-Oncology Biomarker Working Group [33,34]. The evaluation included the assessment of immunocompetent cells at a magnification of 200× in two distinct regions: epithelial and stromal compartments. The average density of certain cells was quantified as a continuous variable by calculating the proportion of the area occupied by immunohistochemically positive cell infiltrates in a specific compartment (either epithelial or stromal) relative to the total epithelial or stromal area. Additionally, density of certain cells was calculated by the number of overall positive cells in whole specimen relative to the number of all viable epithelial cells. A comprehensive evaluation of the tumor region was conducted on each slide, with the exclusion of regions exhibiting ulceration and necrosis from the analysis.

### 4.4. Statistical Analysis

Statistical analysis was performed using MedCalc for Windows, version 19.1 (MedCalc Statistical Software bvba, Ostend, Belgium). Differences in frequency of nominal variables were performed using Fisher’s exact test and χ^2^test. The correlation comparison between PD-L1 and immune cells was calculated using Spearman’s rank correlation analysis. All tests were two-tailed and *p* < 0.05 was considered statistically significant results.

Receiver operating characteristic (ROC) curve analysis was performed to determine optimal cut-off values for biomarkers associated with progression of LD to SCC. Biomarkers were subsequently dichotomized according to the identified cut-off values, and univariate binary logistic regression analysis was used to evaluate their association with progression. Odds ratios (ORs) with corresponding 95% confidence intervals (CIs) were calculated, and the discriminatory performance of each biomarker was assessed using the area under the ROC curve (AUC). Multivariable logistic regression analysis was not performed due to the limited sample size and the low number of progression events (*n* = 9), which would have resulted in an unstable and potentially overfitted model.

## 5. Conclusions

Tumor microenvironment cells could have a possible role in distinguishing laryngeal dysplasia that will progress to laryngeal cancer. The increased presence of tumor-associated macrophages and alterations in T-lymphocyte populations may reflect the development of a local immunosuppressive microenvironment already at the stage of epithelial dysplasia. These observations suggest that immune escape mechanisms may be initiated prior to the development of invasive carcinoma. A better understanding of these early immunological changes could contribute to improved risk stratification of laryngeal dysplasia and may potentially open new perspectives for immune-targeted preventive or therapeutic strategies in premalignant lesions. Further studies, with a larger number of LD specimens are needed.

## Figures and Tables

**Table 1:** Comparison of PD-L1 expression and tumor microenvironment composition in laryngeal lesions.

Variable		Control Group, Median (Range)	LD, Median (Range)	LSCC, Median (Range)	*p* Value
*n* = 179		*n* = 36	*n* = 41	*n* = 102	
Age (years)		40.5 (26.0–78.0)	56.9 (24.7–79.7)	63.0 (43.9–83.6)	<0.0001 *
Sex	female	4 (11.1)	8 (19.5)	7 (6.9)	0.129 ^±^
	male	32 (88.9)	33 (80.5)	95 (93.1)	
CD4	intraepithelial	0 (0.0–2.0)	0.5 (0.0–11.0)	1.0 (0.0–20.0)	<0.0001 *
	stromal	0 (0.0–5.0)	1.5 (0.0–35.0)	9.4 (0.0–40.3)	<0.0001 *
	overall	0.5 (0.0–5.0)	2.6 (0.0–40.0)	10 (0.0–53.3)	<0.0001 *
CD8	intraepithelial	1.0 (0.0–7.0)	2.13 (0.0–14.0)	2.75 (0.0–30.0)	0.002 *
	stromal	1.0 (0.0–8.0)	2.0 (0.0–17.0)	7.05 (0.0–37.3)	<0.0001 *
	overall	2.5 (0.0–12.0)	5.0 (0.0–21.25)	11.1 (0.2–60.0)	<0.0001 *
CD68	intraepithelial	0.0 (0.0–6.0)	0.73 (0.0–11.25)	2.2 (0.0–10.0)	<0.0001 *
	stromal	3.0 (0.0–20.0)	4.0 (0.0–50.0)	7.5 (0.1–30.0)	<0.0001 *
	overall	3.0 (0.0–30.0)	4.0 (0.0–100.0)	9.5 (0.3–50.0)	<0.0001 *
CD163	intraepithelial	0.0 (0.0–2.0)	0.2 (0.0–3.0)	1.5 (0.0–12.0)	<0.0001 *
	stromal	2.0 (0.0–17.5)	5.45 (0.0–60.0)	14.6 (0.0–50.0)	<0.0001 *
	overall	2.0 (0.0–21.5)	5.25 (0.5–100.0)	15.0 (0.0–50.0)	<0.0001 *
CPS, median (range)		0 (0.0–10.0)	0 (0.0–75.0)	7.5(0.0–80.0)	<0.0001 *
TPS, median (range)		0 (0.0–10.0)	0 (0.0–75.0)	2.7(0.0–76.7)	<0.0001 *
CPS	<1	27 (75.0)	23 (56.1)	27 (26.2)	**<0.0001 ^±^**
	1–20	1 (2.8)	8 (19.5)	38 (36.9)	
	≥20	0 (0)	4 (9.8)	29 (28.2)	
	NA	8 (22.2)	6 (14.6)	9 (8.7)	
CPS	<1	27 (75.0)	23 (56.1)	27 (26.2)	**<0.0001 ^±^**
	≥1	1 (2.8)	12 (29.3)	67 (65.0)	
	NA	8 (22.2)	6 (14.6)	9 (8.7)	

* Kruskal–Wallis test, posthoc analysis, is done with Conover test and ^±^ chi-squared test. Abbreviations: CPS—combined positive score; TPS—tumor proportion score; and NA—not applicable (some samples could not be evaluated due to tissue microarray core loss or insufficient tissue; therefore, they were excluded from the analysis). Bold values are statistically significant.

**Table 2:** Comparison of PD-L1 expression and tumor microenvironment composition between low- and high-grade dysplasia.

Variable		Low Grade Dysplasia, Median (Range)	High Grade Dysplasia, Median (Range)	*p* Value
*n* = 41		*n* = 19	*n* = 22	
CD4	Intraepithelial	0.2 (0.0–3.0)	0.85 (0.0–11.0)	**0.025 ^¶^**
	Stromal	0.5 (0.0–5.5)	5.0 (0.0–35.0)	**0.002 ^¶^**
	Overall	0.7 (0.0–5.15)	6.5 (0.0–40.0)	**0.003 ^¶^**
CD8	Intraepithelial	1.88 (0.0–10.0)	3.0 (0.5–14.0)	0.224 ^¶^
	Stromal	1.0 (0.0–8.0)	3.5 (0.25–17.0)	**0.019 ^¶^**
	Overall	3.0 (0.0–15.0)	6.0 (1.5–21.25)	0.066 ^¶^
CD68	Intraepithelial	0.53 (0.0–4.0)	0.85 (0.0–11.25)	0.703 ^¶^
	Stromal	2.63 (0.0–7.0)	4.5 (0.0–50.0)	0.079 ^¶^
	Overall	2.13 (0.0–10.0)	4.75 (0.3–100.0)	0.078 ^¶^
CD163	Intraepithelial	0.2 (0.0–0.7)	0.2 (0.0–3.0)	0.502 ^¶^
	Stromal	4.0 (0.0–15.0)	7.5 (2.5–60.0)	**0.015 ^¶^**
	Overall	3.0 (0.5–17.0)	5.5 (1.0–100.0)	0.199 ^¶^
CPS median		0.0 (0.0–7.0)	0.5 (0.0–75.0)	0.061 ^¶^
TPS median		0.0 (0.0–7.5)	0.3 (0.0–75.0)	0.108 ^¶^
CPS	<1	12 (63.2)	11 (50.0)	0.476 ^§^
	≥1	4 (21.1)	8 (36.4)	
	NA	3 (15.8)	3 (13.6)	
CPS	<1	12 (63.2)	11 (50.0)	0.149 ^±^
	1–20	4 (21.1)	4 (18.2)	
	≥20	0 (0)	4 (18.2)	
	NA	3 (15.8)	3 (13.6)	

^¶^ Mann–Whitney test; ^§^ Fisher’s exact test; and ^±^ chi-squared test. Abbreviations: CPS—combined positive score; TPS—tumor proportion score. Bold values are statistically significant; NA—not applicable (some samples could not be evaluated due to tissue microarray core loss or insufficient tissue; therefore, they are excluded from the analysis).

**Table 3:** Correlation of CPS and TPS with intraepithelial and stromal CD4, CD8, CD68 and CD163 infiltration in precancerous lesions.

			CD4	CD8	CD68	CD163
Intraepithelial	CPS	r_s_	0.107	0.100	0.156	**0.356**
		*p*	0.558	0.579	0.387	**0.049**
	TPS	r_s_	0.047	0.158	0.159	**0.439**
		*p*	0.798	0.379	0.378	**0.013**
			**CD4**	**CD8**	**CD68**	**CD163**
Stromal	CPS	r_s_	0.259	**0.402**	0.226	0.067
		*p*	0.153	**0.020**	0.207	0.719
	TPS	r_s_	0.090	0.323	0.090	0.031
		*p*	0.623	0.066	0.617	0.869

Abbreviations: CPS—combined positive score; TPS—tumor proportion score. Bold values are statistically significant.

**Table 4:** Comparison of expression of investigated markers in dysplasia with and without progression to carcinoma.

Variable		Dysplasia Without Progression	Dysplasia with Progression	*p* Value
*n* = 41		*n* = 32	*n* = 9	
Age(years); median (range)		56.1(24.7–79.7)	61.9 (46.2–78.8)	0.244 ^¶^
Sex	Female	8 (25.0)	0 (0)	0.164 ^§^
	Male	24 (75.0)	9 (100)	
Grade	Low	17 (53.1)	2 (22.2)	0.140 ^§^
	High	15 (46.9)	7 (77.8)	
CPS	<1	18 (56.2)	5 (55.6)	0.679 ^±^
	1–20	5 (15.6)	3 (33.3)	
	≥20	3 (9.4)	1 (11.1)	
	NA	6 (18.8)	0 (0)	
CPS	<1	18 (56.2)	5 (55.6)	0.685 ^§^
	≥1	8 (25.0)	4 (44.4)	
	NA	6 (18.8)	0 (0)	
Variable		Median (Range)	Median (Range)	
CPS		0.0 (0.0–75.0)	0.5 (0.0–25.0)	0.329 ^¶^
TPS		0.0 (0.0–75.0)	0.3 (0.0–25.0)	0.456 ^¶^
CD4	Intraepithelial	0.25 (0.0–11.0)	0.5 (0.0–11.0)	0.223 ^¶^
	Stromal	1.38 (0.0–35.0)	5.0 (0.0–10.00)	0.504 ^¶^
	Overall	2.3 (0.0–17.5)	7.0 (0.0–40.0)	0.293 ^¶^
CD8	Intraepithelial	2.0 (0.0–11.0)	3.0 (0.5–14.0)	0.754 ^¶^
	Stromal	2.0 (0.0–17.0)	4.0 (0.5–10.0)	0.469 ^¶^
	Overall	5.0 (0.0–21.25)	5.0 (2.0–20.0)	0.611 ^¶^
CD68	Intraepithelial	1.0 (0.0–11.25)	0.3 (0.0–1.0)	0.078 ^¶^
	Stromal	4.0 (0.0–12.0)	4.0 (0.1–50.0)	0.583 ^¶^
	Overall	4.5 (0.0–17.0)	2.25 (0.1–100.0)	0.204 ^¶^
CD163	Intraepithelial	0.2 (0.0–3.0)	0.1 (0.0–2.0)	0.733 ^¶^
	Stromal	5 (0.0–25.0)	9.5 (1.0–60.0)	0.070 ^¶^
	Overall	5 (0.5–17.0)	6.25 (0.5–100.0)	0.571 ^¶^

^¶^ Mann–Whitney test; ^§^ Fisher’s exact test; and ^±^ chi-squared test. Abbreviations: CPS—combined positive score; TPS—tumor proportion score; NA—not applicable (some samples could not be evaluated due to tissue microarray core loss or insufficient tissue; therefore, they are excluded from the analysis).

**Table 5:** Factors associated with LD progression.

Factors		OR	95% CI	*p* Value	AUC
CD4	intraepithelial > 0.2	8.00	0.86–74.21	0.067	0.694
	stromal > 4.3	3.75	0.75–18.7	0.103	0.653
	**overall > 6**	**6.25**	**1.14–34.12**	**0.034**	**0.694**
CD8	intraepithelial > 2.5	1.88	0.4–8.74	0.423	0.578
	stromal > 0.3	NA	NA	0.981	0.620
	overall > 7.5	2.06	0.43–9.97	0.370	0.582
CD68	**intraepithelial ≤ 0.5**	**0.161**	**0.03–0.95**	**0.043**	**0.709**
	stromal > 0	NA	NA	0.998	0.600
	**overall ≤ 3**	**0.161**	**0.03–0.95**	**0.043**	**0.709**
CD163	intraepithelial ≤ 0.4	0.429	0.04–4.23	0.468	0.563
	**stromal > 7.5**	**8.33**	**1.39–49.88**	**0.020**	**0.729**
	overall > 4.5	2.54	0.42–15.21	0.308	0.604
CPS (<1, ≥1)		1.80	0.38–8.54	0.459	0.568
CPS > 0 (median)		2.73	0.56–13.37	0.216	0.622
TPS > 0 (median)		2.36	0.5–11.05	0.275	0.605

Odds ratio is calculated based on cut-offs and consequently classified into higher and lower values. Abbreviations: CPS: combined positive score; TPS: tumor proportion score; OR: odds ratio; AUC: area under the curve; and NA: not applicable. Bold values are statistically significant.

## Data Availability

The original contributions presented in this study are included in the article material. Further inquiries can be directed to the corresponding author.
